# Fostering Healthier Interpersonal Dynamics: A Mentalization-Based Antibullying Program for ICU Nurses

**DOI:** 10.1155/jonm/3192349

**Published:** 2025-06-30

**Authors:** Sun Joo Jang, Eunhye Kim, Haeyoung Lee

**Affiliations:** ^1^College of Nursing & The Research Institute of Nursing Science, Seoul National University, Seoul, Republic of Korea; ^2^Department of Nursing, Seoul National University Hospital, Seoul, Republic of Korea; ^3^Red Cross College of Nursing, Chung-Ang University, Seoul, Republic of Korea

**Keywords:** intensive care unit, mentalization, nurse, personality, workplace bullying

## Abstract

**Aim:** This study evaluated the effectiveness of a mentalization-based antibullying program (MBAP) to enhance intensive care unit (ICU) nurses' interpersonal competences.

**Background:** Mentalization is the ability to focus on and understand the mental states of oneself and others. Previous research on ICU nurses has identified mentalization as a critical factor influencing both victimization and perpetration of workplace bullying.

**Methods:** A quasiexperimental design was utilized. A four-session MBAP (with weekly 90-min sessions) aimed at improving awareness of personality traits, interpersonal cognition, and self-conscious emotions was developed. The intervention group underwent the program for a month in June 2023 in four different subgroups. Twenty-six participants were initially recruited for each of the intervention and control groups; however, five intervention group participants withdrew during the allocation phase, with no control group dropouts.

**Results:** Intervention group participants showed a significant reduction in the narcissistic vulnerability subscale of pathological narcissism compared to the control group. They also exhibited a significant decrease in interpersonal cognitive distortions and an increase in mentalization scores. Regarding self-conscious emotions, the shame score significantly decreased, while the guilt score significantly increased.

**Conclusions:** MBAP can be an effective intervention for addressing workplace bullying. By targeting psychological and cognitive factors, including narcissistic vulnerability, interpersonal cognitive distortions, and self-conscious emotions, MBAP holds promise for promoting healthier interpersonal dynamics and emotional regulation among ICU nurses.

**Implications for Nursing Management:** The MBAP can be implemented broadly to prevent workplace bullying and promote a positive organizational culture in ICU settings. Future research should refine and adapt the program to accommodate the unique work characteristics and interpersonal dynamics of nurses in various settings. Workplace bullying is a deeply entrenched negative organizational issue in nursing, and this program could be a highly effective intervention across diverse healthcare environments.

**Trial Registration:** The Clinical Research Information Service (CRIS): KCT0008704

## 1. Introduction

Workplace bullying is a significant global issue with detrimental effects on both individuals and organizations [1]. Nurses, in particular, face high levels of workplace bullying due to their work's demanding and high-pressure nature, compounded by rigid organizational cultures and unsupportive environments [2, 3]. Intensive care unit (ICU) nurses are especially vulnerable given the complexities of their work environment, which involve high-acuity patients, life-support systems, and advanced medical devices, often restricting communication and creating additional stress due to emergencies and heavy workloads [4]. ICUs are inherently challenging and stressful settings [5]. Such conditions expose ICU nurses to significant mental health risks [6], highlighting the urgent need for interventions and policy changes to address these challenges [7].

Workplace bullying poses severe risks to nurses' physical and mental health [8], increases job-related stress, and contributes to turnover [9]. It also undermines productivity and creativity in nursing tasks [8], disrupts team communication and collaboration [10], and ultimately compromises patient safety [1, 8], thus hindering the growth of individual nurses and organizations. Given the various detrimental consequences, developing effective strategies to prevent workplace bullying and mitigate its impact on nurses, patients, and healthcare organizations is critical. Programs addressing both the psychological and emotional aspects of bullying for victims and perpetrators alike are particularly necessary to address the multifaceted nature of workplace bullying.

Mentalization refers to the ability to understand one's own and others' mental states, including desires, intentions, thoughts, feelings, and behaviors [11]. It involves cognitive processes that focus on and interpret the mental states of oneself and others [12]. Mentalization plays a crucial role in emotional regulation, interpersonal relationships, and self-awareness, serving as a core mechanism for social functioning [11, 13]. Previous research on ICU nurses identified mentalization as a key predictor of both victimization and perpetration of workplace bullying [14]. Lower levels of mentalization are associated with difficulties in interpersonal relationships due to a lack of understanding of oneself and others [15]. Conversely, higher mentalization levels are linked to greater life satisfaction and reduced depression and anxiety [16, 17]. High workplace stress can compromise mentalization, making nurses with lower mentalization levels more susceptible to experiencing or perpetrating workplace bullying. Since mentalization-based treatment has been utilized in psychological therapies for conditions such as borderline personality disorder [13, 18], a mentalization-based antibullying program (MBAP) could serve as a potential approach for preventing workplace bullying in ICU nurses.

Among the various predictors of workplace bullying, understanding personality traits linked to bullying is essential [19]. Narcissism is a personality trait categorized into normal narcissism, which is typical in developmental stages, and pathological narcissism, which leads to maladaptive behaviors in daily life [20]. Pathological narcissism has been identified as a significant predictor of workplace bullying [21]. A meta-analysis of the relationship between narcissism and aggression demonstrated a significant association between the two [20]. Narcissistic personality is multidimensional, encompassing narcissistic grandiosity and narcissistic vulnerability [20, 22]. Narcissistic grandiosity is characterized by entitlement and exploitative or arrogant behaviors, while narcissistic vulnerability involves a strong need for admiration and sensitivity to criticism, often leading to frustration when such needs are unmet [22]. Pathological narcissism has been linked to workplace bullying [14, 21], and mentalization-based treatment can be an effective intervention for managing pathological narcissism [23].

Self-conscious emotions such as shame and guilt are cognitive and emotional constructs that encourage socially desirable behaviors while discouraging undesirable ones [24]. Shame and guilt are distinguished based on whether their focus is on the self or on one's actions [25]. Specifically, guilt is characterized by a focus on one's behavior, while shame centers on the self [25]. In addition, individuals with a high tendency toward shame, referred to as shame-proneness, are more likely to blame others; thus, it is possible to reduce bullying by identifying and addressing shame [26]. Self-conscious emotions encompass four distinct features: shame-proneness, guilt-proneness, detachment/unconcern, and externalization [27]. Detachment/unconcern reflects a tendency to emotionally disengage or remain indifferent in situations that typically evoke self-conscious emotions such as shame or guilt [27]. Externalization refers to the inclination to attribute emotional experiences to external causes rather than internal ones [27]. In this study, we included all four features for measuring self-conscious emotions.

Interpersonal cognitive distortion has been identified as a potent predictor of workplace bullying in addition to pathological narcissism in nurses [21]. Interpersonal cognitive distortion influences role performance by shaping expectations about others' roles and one's responsibilities in fulfilling expected roles [28]. Rigid schemas resulting from these distortions can lead to misunderstandings in various interpersonal relationships [29]. For example, new nurses in established nursing environments often perceive themselves as targets of workplace bullying simply because they are new [2]. Given the critical importance of collegiality and solidarity among nurses to ensure positive interpersonal relationships in nursing practice [30], addressing interpersonal cognitive distortion may help prevent and mitigate negative relationships, as exemplified by workplace bullying. Kürümlüoğlugil and Tanriverdi [31] emphasized the potential of individual psychoeducation in modifying interpersonal cognitive distortions.

Against this backdrop, this study developed and evaluated a MBAP in consideration of individual personality traits related to workplace bullying (i.e., pathological narcissism, self-conscious emotions, and interpersonal cognitive distortion) among ICU nurses. We believe that this program will help ICU nurses better understand the personality traits of themselves and others, enabling them to regulate behaviors that could negatively impact interpersonal relationships, ultimately helping to prevent workplace bullying.

The study hypotheses are as follows:

  H1: The intervention group that participated in the MBAP will exhibit decreased narcissistic grandiosity after the program compared to the control group.
  H2: The intervention group that participated in the MBAP will exhibit decreased narcissistic vulnerability after the program compared to the control group.
  H3: The intervention group that participated in the MBAP will exhibit decreased interpersonal cognitive distortion after the program compared to the control group.
  H4: The intervention group that participated in the MBAP will exhibit increased mentalization after the program compared to the control group.
  H5: The intervention group that participated in the MBAP will exhibit decreased shame (self-conscious emotion) after the program compared to the control group.
  H6: The intervention group that participated in the MBAP will exhibit increased guilt (self-conscious emotion) after the program compared to the control group.
  H7: The intervention group that participated in the MBAP will exhibit decreased detachment (self-conscious emotion) after the program compared to the control group.
  H8: The intervention group that participated in the MBAP will exhibit decreased externalization (self-conscious emotion) after the program compared to the control group.
  H9: The intervention group that participated in the MBAP will exhibit decreased workplace bullying–victim aspect after the program compared to the control group.
  H10: The intervention group that participated in the MBAP will exhibit decreased workplace bullying–perpetrator aspect after the program compared to the control group.

## 2. Methods

### 2.1. Research Design

This study utilized a quasiexperimental design (nonequivalent control group pretest–posttest design).

### 2.2. Research Participants and Data Collection

This study enrolled ICU nurses based on specific inclusion and exclusion criteria, as follows: (1) Inclusion criteria: Nurses working in the ICU at a tertiary hospital with a minimum of 3 months of employment at the time of the study, who provided informed consent to participate, and (2) Exclusion criteria: Nurses in a probationary period and nurses not directly involved in patient care (e.g., administrators, educators, and unit managers).

The minimum sample size was estimated using G^∗^power Version 3.1.9.7 software. Considering a significance level of 0.05, a power of 0.95, three measurements on RM-ANOVA, and a medium effect size of *f* = 0.25, the minimum sample size was calculated to be 21 for each group, totaling 42. Accounting for a 20% dropout rate, the target sample size was set at 52, with 26 in each group. Referring to a previous study indicating that predictors of workplace bullying differ before and after “3 years of employment” [21], we stratified the length of career into 3 years or less and more than 3 years. Participants were allocated to the intervention and control groups at an equal ratio, with 26 in each group.

**Figure 1:**
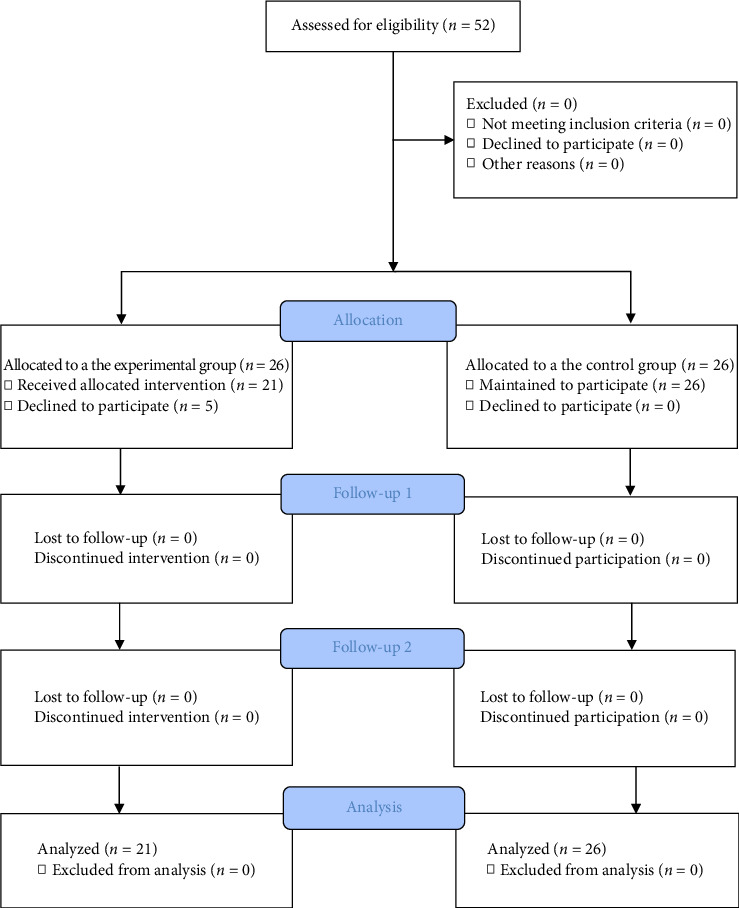
Study flow diagram.

Recruitment notices were posted on bulletin boards in the nurses' lounge in the ICU or sent via the hospital's groupware email between March and June 2023. Intervention group participants were recruited between March and April 2023, while those wishing to participate in the control group were recruited between May and June 2023. Stratification was based on experience using a three-year criterion. Interested participants could freely apply or withdraw their application for the study through a Google application link or QR code. Five out of 26 intervention group participants withdrew their application during the assignment phase, while there were no dropouts in the control group. Participants in both the intervention and control groups responded to the survey via a Google survey link at the start, after 2 weeks, and after 4 weeks. Data from 21 participants in the intervention group and 26 participants in the control group were used for the final analysis (Figure 1).

### 2.3. Research Tools

The MBAP used as the study intervention was developed by the authors with the goal of promoting “accurate recognition of one's personality traits, interpersonal cognition, and self-conscious emotions.” The program comprises four weekly sessions, each lasting 90 min. The topics and content of each session are summarized in Table 1. The topics, content, and methods of the MBAP were reviewed by seven experts, including three psychologists, two mental health nursing professors, and two mental health advanced practice nurses. The average item content validity index was 0.98, and the scale's content validity universal agreement index was 0.83.

The intervention group was divided into four subgroups for different time slots: mornings or afternoons on Wednesdays or Thursdays in June 2023. Each group comprised six to seven participants, considering the shift work schedule and total clinical experience. Participants were instructed to attend their assigned days but were allowed to attend another group once for unavoidable reasons, such as sudden work changes. An administrative nurse not involved in the study served as a research assistant. The administrative nurse held discussions with ICU nurse participants in the intervention group to adjust their eight-hour rotation duties so they could participate in the program.

Measures were implemented to reduce the likelihood of assigning participants who might be uncomfortable with each other due to workplace bullying issues in the same group. This included dividing participants into two groups based on the 3-year experience criterion, assigning subgroups to different nursing units at work, and grouping nurses with similar overall experience. For the control group, three rounds of surveys were conducted every 2 weeks from July to August.

To ensure consistency in program delivery, all sessions for the four subgroups were conducted exclusively by the first author, who is a professor specializing in psychiatric nursing as well as a candidate for Jungian psychoanalysis and a psychiatric mental health nurse practitioner. With 15 years of clinical experience as a clinical specialist nurse in adult mental health nursing and a unit manager on a psychiatric ward, she is particularly skilled in leading group therapy programs.

### 2.4. Measurement Tool

Based on a previous study concerning participants' general characteristics and work-related characteristics [14], we developed a self-report questionnaire for data collection. The instruments described in the following were used to assess self-conscious emotion, mentalization, and workplace bullying (both victim and perpetrator aspects), after obtaining permission from the original developers and authors of the Korean version. The reliability and validity of all instruments were verified in the Korean adult population.

#### 2.4.1. Pathological Narcissism

Pathological narcissism was assessed using the Korean version [32] of the Pathological Narcissism Inventory [33], comprising 35 items that distinguish between narcissistic grandiosity and narcissistic vulnerability. Each item is rated on a seven-point Likert scale (0 = not at all to 6 = very likely), with scores ranging from 0 to 210, where a higher score indicates more severe pathological narcissism. The Cronbach's α for the Pathological Narcissism Inventory was 0.95 and 0.78–0.93 for the factors in the study by Pincus et al. [33] and 0.92 (0.85–0.92) in the Korean version. In this study, the Cronbach's α was 0.95 (0.90–0.93).

#### 2.4.2. Self-Conscious Emotion

Self-conscious emotions were measured using the Test of Self-Conscious Affect Version 3 Short (TOSCA-3S) [27], which measures four distinct tendencies (shame-proneness, guilt-proneness, detachment/unconcern, and externalization) in 11 negative scenarios that are commonly encountered in everyday life. Each tendency is rated on a five-point Likert-type scale (1 = not likely and 5 = very likely) with 11 items. There is one item measuring each of the four areas for each scenario, resulting in a total of 44 items with 11 items for each area. Higher scores indicate a greater proneness to shame, guilt, detachment/unconcern, and externalization (range: 11–55) [27]. The Cronbach's α for the TOSCA-3S was reported to range from 0.77 to 0.88 [27]. In this study, Cronbach's α ranged from 0.70 to 0.73.

#### 2.4.3. Interpersonal Cognitive Distortion

The Korean version of the Interpersonal Cognitive Distortions Scale developed by Hamamci and Buyukozturk [34] and adapted by Lee et al. [15] was used to measure the degree of interpersonal cognitive distortions. It comprises 19 items and three factors (interpersonal rejection, unrealistic relationship expectations, and interpersonal misperception) and employs a five-point Likert-type scale (0 = not at all and 4 = very much). Higher scores indicate higher levels of interpersonal cognitive distortions (range: 0–76). The Cronbach's α was 0.67 in the study by Hamamci and Buyukozturk ([34], 0.80 (0.65–0.82) in the Korean version [15], and 0.75 in this study.

#### 2.4.4. Mentalization

The Korean version [15] of the Mentalization Scale [16] was employed to measure the degree of mentalization. This tool comprises 28 items and three domains: self-related mentalization, other-related mentalization, and motivation to mentalize. Each item is rated on a five-point Likert-type scale (1 = completely disagree and 5 = completely agree). The Cronbach's α was 0.84 (0.74–0.79) for the original tool [16]), 0.88 (0.74–0.84) for the Korean version, and 0.80 in this study.

#### 2.4.5. Workplace Bullying (Victim and Perpetrator Aspects)

The victim aspect of workplace bullying was measured using the Korean version of the Negative Acts Questionnaire–Revised (NAQ-R) [35] adapted by Nam et al. [36]. It comprises 22 items on a five-point Likert-type scale, with scores ranging from 22 to 110. The Cronbach's α was 0.93 at the time of development [35], 0.93 in the Korean version [36], and 0.90 in this study.

The perpetrator aspect of workplace bullying was measured using 22 items from the NAQ-Perpetrator, which were modified and validated from the NAQ-R [35] for measuring the perpetrator's perspective. Each item is rated on a five-point Likert-type scale, with scores ranging from 22 to 110. The Cronbach's α was 0.97 at the time of the tool's development and 0.89 in this study.

### 2.5. Ethical Considerations

Due to the participants being vulnerable individuals who are subordinates of one of the authors, additional protective measures were implemented to ensure no coercion or undue influence on their participation. Only the author from an external institution was in contact with the participants and handled participant-related information. The co-author was completely blocked from interacting with the participants or accessing participant-related information, and all program sessions were conducted solely by the corresponding author from an external institution. This approach minimized potential risks, discomfort, or harm to the research participants.

The study was conducted after receiving approval from the institutional review board of the hospital where the participating nurses work (no. H-2302-073-1405, initial approval date: April 5, 2023). Participants who accessed the study through the posted study recruitment post were provided with an explanation of the study purpose and methods, assurance of anonymity, and the option to withdraw participation at any time. Informed consent was obtained from those who voluntarily wished to participate in the study.

### 2.6. Data Analysis

Data were analyzed using the SPSS Statistics program 27.0. The baseline homogeneity of the participants' general characteristics, work-related characteristics, and prescores of dependent variables was tested using independent *t*-tests and *x*^2^ tests. Based on the recommendations of a previous study [37] for using generalized estimating equations (GEEs) for repeated measurements, clustered, and correlated data that can adjust for the effects of confounders without assuming independence of variance and homogeneity, we conducted GEE with an autoregressive correlation structure to examine changes before, during, and after the program (measured at three time points: before the program, 2 weeks later, and immediately after the program's conclusion). Variables that showed differences in the baseline homogeneity test between the intervention and control groups were controlled in the GEE model. We used a per-protocol analysis, which analyzes the data of only those individuals who are retained in the allocated group and complete the trial. The reliability of each tool was verified by calculating Cronbach's α.

## 3. Results

### 3.1. Homogeneity Test of Participants' Characteristics and Dependent Variables

The mean age of the participants was 30.04 years (SD: 4.07), and the mean ICU career was 6.28 years (SD: 4.01). All 21 intervention group participants were women; however, six out of 26 (23.1%) in the control group were men. There were no significant differences in the general characteristics (other than gender), work-related characteristics (Table 2), or dependent variables at baseline between the two groups (Table 3).

### 3.2. Effects of MBAP on Participants' Pathological Narcissism, Interpersonal Cognitive Distortion, and Mentalization

The intervention group exhibited a lower score in pathological grandiosity compared to the control group; however, this difference was not significant; thus, Hypothesis 1 was rejected (Wald's test = 0.06, *p*=0.912). However, pathological vulnerability was significantly lower in the intervention group, supporting Hypothesis 2. As shown in Table 4, GEE analysis showed that the intervention group demonstrated a 6.75-point reduction in pathological vulnerability after the program compared to the control group (Wald's test = 4.42. *p*=0.026).

The intervention group also exhibited a significant decrease in interpersonal cognitive distortion compared to the control group, supporting Hypothesis 3. GEE analysis revealed no significant main effects of group or time, although a significant interaction between group and time (Wald's test = 5.04, *p*=0.025) was observed. The intervention group showed a 3.41-point decrease in interpersonal cognitive distortion at the midpoint of the program (2 weeks) and a 4.70-point decrease after the program's conclusion (Table 4).

**Figure 2:**
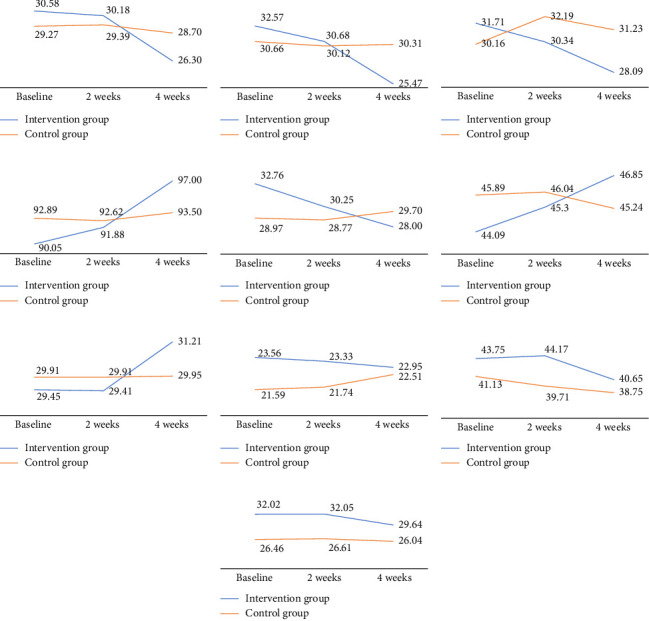
Outcome changes in group comparison; the effect of the MBAP on (a), (b), (c), (d), (e), (f), (g), (h), (i), and (j). (a) Narcissistic grandiosity. (b) Narcissistic vulnerability. (c) Interpersonal cognitive distortion. (d) Mentalization. (e) Shame. (f) Guilt. (g) Detachment. (h) Externalization. (i) Victim aspect. (j) Perpetrator aspect.

Furthermore, the intervention group exhibited a significant increase in mentalization scores compared to the control group, supporting Hypothesis 4. GEE analysis revealed no significant main effects of group or time, although a significant interaction between group and time (Wald's test = 5.04, *p*=0.025) was observed. The intervention group exhibited a 6.34-point increase in mentalization scores after the program compared to the control group (Table 4 and Figure 2).

### 3.3. Effects of MBAP on Participants' Self-Conscious Emotion

The intervention group showed a significant decrease in shame scores and a significant increase in guilt scores compared to the control group, supporting Hypotheses 5 and 6. GEE analysis revealed no significant main effects of group or time for shame (Wald's test = 9.80, *p*=0.002) and guilt (Wald's test = 6.58, *p*=0.010), although a significant interaction between group and time was observed. The intervention group showed a 5.49-point decrease in shame scores and a 3.42-point increase in guilt scores after the program compared to the control group (Table 5 and Figure 2). However, no significant differences were observed in changes in detachment and externalization between the intervention and control groups, meaning Hypotheses 7 and 8 were rejected.

### 3.4. Effects of MBAP on Participants' Workplace Bullying–Victim and Perpetrator Aspects

The workplace bullying–victim aspect score and perpetrator aspect score were lower in the intervention group than in the control group, however, not to a significant extent. Thus, Hypothesis 9 (Wald's test = 0.05, *p*=0.832) and Hypothesis 10 (Wald's test = 1.01, *p*=0.315) were rejected (Table 6 and Figure 2).

## 4. Discussion

This study examined the effectiveness of a MBAP in preventing workplace bullying among ICU nurses. The program was designed to enhance awareness of personal traits, interpersonal cognition, and self-conscious emotions. It comprised three key components: resolving conflict, which encouraged participants to explore their own and others' mental states; expressing oneself, which helped differentiate thoughts from emotions; and enhancing interpersonal cognitive competence, which focused on practicing empathetic listening, speaking, and mentalizing. The findings regarding changes observed at two and 4 weeks postintervention are discussed as follows.

The intervention group exhibited a reduction in narcissistic grandiosity compared to the control group, although this change was not statistically significant. However, a significant reduction in narcissistic vulnerability was observed. As shown in Figure 2, GEE analysis revealed a consistent and sustained decrease in narcissistic vulnerability. This progressive decline reflects the cumulative effect of the MBAP, underscoring the importance of continued participation in the program. A previous study identified narcissistic vulnerability as the primary predictor of the victimization aspect of workplace bullying among ICU nurses [14]. High levels of narcissistic vulnerability are associated with feelings of shame when external validation is lacking [38], as well as unrealistic expectations for wanting only positive responses from others, which can lead to negative emotional outcomes such as depression [39]. Thus, nurses with heightened narcissistic vulnerability are more prone to misinterpreting negative feedback or criticism as bullying, suggesting that reducing narcissistic vulnerability is essential for mitigating workplace bullying. Interventions aimed at improving emotional regulation and self-esteem are particularly valuable for nurses with high narcissistic vulnerability [21, 32]. In addition, increasing awareness of narcissism is critical to mitigate the potential harm caused by narcissistic behaviors to colleagues and the organization [40]. This study demonstrated that the developed MBAP could serve as an effective intervention for addressing narcissistic vulnerability, ultimately reducing the risk of workplace bullying.

The intervention group also exhibited a significant reduction in interpersonal cognitive distortion compared to the control group. While the main effects of group and time were not statistically significant in the GEE analysis, the group and time interaction effect was significant, with the intervention group exhibiting a consistent downward trend in interpersonal cognitive distortion. This trend not only supports the statistical findings but also suggests that participants internalized cognitive changes facilitated by MBAP. Reducing interpersonal cognitive distortions is critical for fostering long-term improvements in interpersonal relationships in the high-pressure ICU environment. Alongside pathological narcissism, interpersonal cognitive distortion has been identified as a predictor of workplace bullying, even when controlling for organizational culture, work-related characteristics, and demographic variables [21]. Interpersonal cognitive distortions are associated with workplace bullying in nurses, and mentalization-based interventions have been shown to enhance emotional regulation and interpersonal relationships [23]. In this study, the MBAP yielded a significant decrease in interpersonal cognitive distortions. Previous studies have also shown that interventions such as mindfulness practices combined with cognitive approaches [41] and individual psychoeducation [31] can effectively address interpersonal cognitive distortions.

The intervention group's participants demonstrated a significant increase in mentalization scores compared to the control group. While the GEE analysis did not show significant main effects of group or time, a significant group and time interaction effect was observed. As shown in Figure 2, mentalization scores in the intervention group increased consistently throughout the study period, which reflects the effectiveness of the program in fostering reflective abilities, a critical skill for managing complex interpersonal dynamics and mitigating workplace bullying.

A previous study identified mentalization as a key factor influencing both victimization and perpetration aspects of workplace bullying among ICU nurses [14], that is, lower levels of mentalization were associated with higher scores in both the victim and perpetrator dimensions of workplace bullying, suggesting that enhancing mentalization could reduce these behaviors. The consistent improvement in mentalization scores observed in the intervention group represents a markedly positive outcome. Since mentalization is a mental activity that involves focusing on and comprehending the minds of oneself and others [12, 23], higher levels of mentalization would allow for a better understanding of oneself and others and prevent potential issues potentially arising in interpersonal relationships.

The intervention group's participants also showed significant changes in self-conscious emotions, with a significant decrease in shame scores and an increase in guilt scores compared to the control group. GEE analysis indicated no significant main effects of group or time, but a significant group and time interaction effect. While no significant changes were observed for detachment or externalization between the two groups, the changes in shame and guilt followed distinct patterns. In the intervention group, shame decreased markedly, while guilt increased steadily, suggesting a shift from maladaptive self-critical emotions toward more constructive moral emotions. As previously discussed, guilt focuses on specific behaviors rather than devaluing the self, unlike shame, which is characterized by self-deprecating tendencies [25]. Shame-proneness is significantly associated with anxiety symptoms [42] and negative psychological outcomes, such as depression and anxiety [25]. Beyond being mere feelings, shame and guilt serve an adaptive function by motivating individuals to avoid harmful actions and maintain interpersonal trust [43]. In the highly stressful ICU setting, managing these emotions appropriately is critical for fostering positive interpersonal relationships among nurses. Alvarez [44] emphasized that addressing shame and enhancing resilience against it can alleviate negative emotions such as depression. Similarly, Merkin [26] suggested that shame-prone individuals tend to blame others, meaning that identifying and remediating shame in these individuals could help reduce bullying. The significant reduction in shame observed in the intervention group highlights the potential of the MBAP as an effective intervention for reducing negative emotions and workplace bullying in ICU nurses.

Although the intervention group showed reduced workplace bullying–victim and perpetrator aspects scores compared to the control group, these changes were not statistically significant. This pattern suggests that, while psychological and cognitive factors may improve, sustained interventions or supplemental strategies may be required to achieve measurable changes in workplace bullying behaviors. Extending the intervention duration and incorporating long-term follow-up may be necessary to better understand and effectively influence workplace bullying behaviors.

This study has several limitations. First, the sample was relatively small and the participants were drawn exclusively from ICU nurses at a single institution, possibly limiting the generalizability of the findings. To enhance generalizability, future studies should include larger samples and diverse healthcare settings. Second, the intervention period may not have been long enough to observe behavioral changes related to workplace bullying. Extending the intervention duration or conducting additional evaluations or longitudinal studies could provide further insight into the long-term effects of a MBAP on workplace bullying. Third, while the self-reported measures used in this study were validated, the potential impact of biases, such as social desirability or recall bias, cannot be completely ruled out. Fourth, differences in gender distribution between the intervention and control groups may have introduced a bias. Future studies should ensure gender-homogeneous groups to confirm the effects of the intervention more accurately. Fifth, the instruments were not originally validated within the specific context of ICU nursing. Further research is needed to confirm their validity among nurse populations. Finally, this study focused on individual personality traits, however, environmental factors such as organizational culture can also significantly influence nurses' interpersonal relationships and job performance. Thus, future studies should consider integrating these contextual factors into intervention planning and evaluation.

Despite these limitations, this study is significant as it developed and evaluated the effectiveness of a MBAP to prevent workplace bullying. The changes observed in the GEE analyses provide valuable insights into the cumulative effects of the program over time and reinforce the quantitative evidence supporting its efficacy. Moreover, the findings emphasize the need for more intensive interventions to translate these improvements into actual reductions in workplace bullying experiences.

## 5. Conclusion

This study demonstrated that a MBAP is an effective intervention for addressing workplace bullying in high-stress environments such as ICUs. By targeting psychological and cognitive factors, including narcissistic vulnerability, interpersonal cognitive distortions, and self-conscious emotions, a MBAP holds promise for promoting healthier interpersonal dynamics and emotional regulation among ICU nurses. Although further research is warranted to address the study's limitations and strengthen its findings, this study provides evidence supporting the potential of mentalization-based interventions to improve workplace culture and nurse well-being in ICU settings. Future research should focus on larger and more diverse samples, extended intervention periods, and long-term evaluations to fully assess and enhance the efficacy of the MBAP. Moreover, incorporating qualitative data in addition to quantitative measures may help future studies better capture the multifaceted impact of the intervention program.

## 6. Implications for Nursing Management

Recognizing and addressing nurses' personality traits through targeted education and interventions are critical in preventing the reinforcement of negative tendencies. Developing and implementing strategies to enhance mentalization skills, helping nurses better understand and interpret their own and others' thoughts and behaviors, should be prioritized. At the organizational level, fostering a culture that integrates individual personality traits into a supportive and positive nursing environment is equally important. These initiatives can strengthen interpersonal relationships and teamwork among nurses, thus improving the quality of patient care and promoting a healthier organizational culture.

Moreover, incorporating psychological interventions such as mentalization-based programs into staff development efforts may contribute to long-term improvements in emotional regulation, communication, and conflict resolution among nurses. Nurse managers can also use insights from this study to identify high-risk individuals, such as those with heightened narcissistic vulnerability or interpersonal distortions, and provide tailored support or mentoring. Expanding such interventions beyond individual-level training toward unit- or systemwide initiatives may further enhance resilience and reduce the prevalence of workplace bullying in high-stress environments such as ICUs.

## Figures and Tables

**Table 1:** Mentalization-based antibullying program.

Session	Themes	Goals	Contents
1	Orientation	To motivate participation and build intimacy and trust among group members To explore one's and each other's minds	1. Program orientation and lecture (30 min)
			2. Mentalization-based approach (60)
			(1) Warming up (30 min)
			- Shame versus guilt
			- Attributional style
			- Interpersonal cognitive distortion
			(2) Mentalization STORM model (20 min)
			(3) Sharing (10 min)

2	Resolving conflict	To explore one's and each other's minds	1. Life sharing (15 min)
			2. Mini lecture on mentalization (15 min)
			3. Mentalization-based approach (60 min)
			(1) Warming up (15 min)
			- Theory of mind
			(2) Improving reflection functioning (35 min)
			- Conflict situations with colleagues
			- Finding own and other's behaviors and motives in a conflict scenario
			(3) Sharing feelings and evaluation (10 min)

3	Expressing myself	To distinguish thoughts and emotions	1. Life sharing (15 min)
			2. Mini lecture on mentalization (15 min)
			3. Mentalization-based approach (60 min)
			(1) Warming up (15 min)
			- Theory of mind
			(2) Improving reflection functioning (35 min)
			- Conflict situations with colleagues
			- Finding own and other's behaviors and motives in a conflict scenario
			(3) Sharing feelings and evaluation (10 min)

4	Enhancing interpersonal cognitive competence	To practice mentalizing To practice empathetic listening and speaking	1. Life sharing (15 min)
			2. Mini lecture on mentalization (15 min)
			3. Mentalization-based approach (60 min)
			(1) Warming up (15 min)
			- Hypermentalizing issues
			- Explicit, external, and internal mentalization
			(2) Improving reflection functioning (35 min)
			- Conflict situations with colleagues
			- Finding own and other's behaviors and motives in a conflict scenario
			(3) Sharing feelings and evaluation (10 min)

*Note:* STORM: security, trauma focus, obtaining skills, resource focus, and mentalization.

**Table 2:** Homogeneity test of participants' characteristics (*N* = 47).

Characteristics	Categories	Int (*n* = 21) *n* (%) Mean (SD)	Cont (*n* = 26) *n* (%) Mean (SD)	Total (*N* = 47) *n* (%) Mean (SD)	*χ* ^2^ or *t*	*p*
Age (years)		30.14 (4.51)	29.96 (3.77)	30.04 (4.07)	0.15	0.881

Gender	Male	0 (0)	6 (23.1)	6 (12.8)	5.56	0.026
	Female	21 (100)	20 (76.9)	41 (87.2)		

Marital status	Single	16 (76.2)	23 (88.5)	39 (83.0)	1.24	0.437
	Married	5 (26.8)	3 (11.5)	8 (17.0)		

Religion	Yes	7 (33.3)	13 (50.0)	20 (42.6)	1.32	0.374
	No	14 (66.7)	13 (50.0)	27 (57.4)		

Education level	Bachelor's degree	20 (95.2)	24 (92.3)	44 (93.6)	10.94	> 0.999
	Master's degree	1 (4.8)	2 (7.7)	3 (6.4)		

Subjective health status	Poor	4 (19.0)	4 (15.4)	8 (17.0)	0.11	0.519
	Good	17 (81.0)	22 (84.6)	39 (83.0)		

Total working years		6.65 (4.20)	5.98 (3.91)	6.28 (4.01)	0.56	0.577

Current unit working years		4.38 (2.54)	3.66 (2.98)	3.98 (2.79)	0.86	0.386

Position	Staff nurse	16 (76.2)	23 (88.5)	39 (83.0)	1.24	0.437
	Charge nurse	5 (23.8)	3 (11.5)	8 (17.0)		

Antibullying education^†^	Yes	10 (47.6)	11 (42.3)	21 (44.7)	0.13	0.774
	No	11 (52.4)	15 (57.7)	26 (55.3)		

Experience of being bullied^†^	Yes	3 (14.3)	3 (11.5)	6 (12.8)	0.08	0.558
	No	18 (85.7)	23 (88.5)	41 (87.2)		

Experience of bullying^†^	Yes	1 (4.8)	0 (0)	1 (2.1)	1.27	0.447
	No	20 (95.2)	26 (100)	46 (97.9)		

Witness of workplace bullying^†^	Yes	9 (42.9)	8 (30.8)	17 (36.2)	0.74	0.543
	No	12 (57.1)	18 (69.2)	30 (63.8)		

*Note:* Int, intervention group; Cont, control group.

^†^Within 1 year.

**Table 3:** Homogeneity test of dependent variables (*N* = 47).

Variables	Int (*n* = 21) *n* (%) Mean (SD)	Cont (*n* = 26) *n* (%) Mean (SD)	Total (*N* = 47) *n* (%) Mean (SD)	*χ* ^2^ or *t*	*p*
Narcissistic grandiosity	29.62 (13.37)	30.04 (13.21)	29.85 (13.14)	0.11	0.915

Narcissistic vulnerability	32.33 (16.98)	30.85 (16.28)	31.51 (16.43)	0.31	0.761

Interpersonal cognitive distortion	31.81 (7.20)	30.08 (8.40)	30.85 (7/85)	0.75	0.458

Mentalization	89.67 (7.53)	93.19 (11.20)	91.62 (9.79)	1.23	0.224

*Self-conscious emotion*					
Shame	32.43 (6.32)	29.23 (5.19)	30.66 (5.88)	1.91	0.063
Guilt	44.24 (3.78)	45.77 (2.57)	45.09 (3.22)	1.58	0.122
Detachment	29.10 (4.57)	30.19 (6.60)	29.70 (5.75)	0.65	0.522
Externalization	23.24 (5.28)	21.85 (5.56)	22.47 (5.43)	0.87	0.388

*Workplace bullying*					
Victim aspect	43.38 (15.15)	41.42 (14.38)	42.30 (14.60)	0.45	0.653
Perpetrator aspect	30.95 (10.07)	27.31 (7.26)	28.94 (8.72)	1.39	0.173

**Table 4:** Effects of MBAP on participants' narcissism, interpersonal cognitive distortion, and mentalization (*N* = 47).

	95% Wald CI					
	*B*	SE	Lower	Upper	Wald *χ*^2^	*p*
(a) Narcissistic grandiosity						
Group^†^						
MBAP	1.31	3.82	−6.17	8.79	0.12	0.732
Time						
Baseline	0	0				
2 weeks	0.12	1.14	−2.11	2.34	0.01	0.919
4 weeks	−0.58	1.54	−3.59	2.44	0.14	0.708
Interaction of groups and time^†^						
Baseline	0	0				
2 weeks	−0.52	2.41	−8.45	1.03	2.36	0.125
4 weeks	−3.71	2.42	−8.45	1.03	0.06	0.912
(b) Narcissistic vulnerability						
Group^†^						
MBAP	1.91	4.93	−7.76	11.58	0.15	0.699
Time						
Baseline	0	0				
2 weeks	−0.54	1.50	−3.48	2.41	0.13	0.720
4 weeks	−0.35	1.70	−3.69	3.00	0.04	0.839
Interaction of groups and time^†^						
Baseline	0	0				
2 weeks	−1.35	2.30	−5.86	3.16	0.34	0.558
4 weeks	−6.75	3.03	0.10	2.82	4.42	0.026^∗^
(c) Interpersonal cognitive distortion						
Group^†^						
MBAP	1.56	2.48	−3.30	6.41	0.639	0.530
Time						
Baseline	0	0				
2 weeks	2.04	1.11	−0.14	4.22	3.36	0.067
4 weeks	1.08	1.09	−1.06	3.21	0.98	0.323
Interaction of groups and time^†^						
Baseline	0	0				
2 weeks	−3.41	1.49	−6.33	−0.50	5.26	0.022^∗^
4 weeks	−4.70	2.09	−8.79	−0.60	5.04	0.025^∗^
(d) Mentalization						
Group^†^						
MBAP	−2.84	2.88	−8.49	2.81	0.97	0.324
Time						
Baseline	0	0				
2 weeks	−0.27	1.48	−3.17	2.63	0.03	0.857
4 weeks	0.62	1.63	−2.58	3.81	0.14	0.705
Interaction of groups and time^†^						
Baseline	0	0				
2 weeks	2.10	1.93	−1.67	5.88	1.19	0.275
4 weeks	6.34	2.18	2.06	10.61	8.44	0.004^∗∗^

*Note:* Covariates: gender and *p* value: GEE model used with adjustment for covariates.

^†^Reference: Control group for the group effect, baseline values for the time effect, and baseline values of the control group for interactions.

^∗^ *p* < 0.05.

^∗∗^ *p* < 0.01.

**Table 5:** Effects of MBAP on participants' self-conscious emotion (*N* = 47).

	95% Wald CI					
	*B*	SE	Lower	Upper	Wald *χ*^2^	*p*
(e) Self-conscious emotion: shame						
Group^†^						
MBGT	3.80	1.68	−0.09	6.48	3.64	0.056
Time						
Baseline	0	0				
2 weeks	−0.19	1.02	−2.20	1.82	0.04	0.851
4 weeks	0.73	1.05	−1.33	2.79	0.48	0.486
Interaction of groups and time^†^						
Baseline	0	0				
2 weeks	−2.32	1.52	−5.31	0.67	2.31	0.128
4 weeks	−5.49	1.75	−8.93	−2.05	9.80	0.002^∗∗^
(f) Self-conscious emotion: guilt						
Group^†^						
MBGT	−1.80	0.99	−3.75	0.14	3.30	0.069
Time						
Baseline	0	0				
2 weeks	0.15	0.67	−1.16	1.47	0.05	0.819
4 weeks	−0.65	0.69	−2.00	0.69	0.90	0.342
Interaction of groups and time^†^						
Baseline	0	0				
2 weeks	1.06	1.19	−1.27	3.40	0.79	0.373
4 weeks	3.42	1.33	0.81	6.03	6.58	0.010^∗^
(g) Self-conscious emotion: detachment						
Group^†^						
MBGT	−0.46	1.78	−3.95	3.03	0.07	0.796
Time						
Baseline	0	0				
2 weeks	0.01	0.05	−0.11	0.11	0.00	> 0.999
4 weeks	0.04	0.93	−1.79	1.87	0.00	0.967
Interaction of groups and time^†^						
Baseline	0	0				
2 weeks	−0.04	0.73	−0.19	0.10	0.36	0.548
4 weeks	1.72	1.31	−0.84	4.29	1.74	0.187
(h) Self-conscious emotion: externalization						
Group^†^						
MBGT	1.98	1.73	−1.41	5.36	1.31	0.252
Time						
Baseline	0	0				
2 weeks	0.15	0.56	−0.94	1.25	0.08	0.783
4 weeks	0.92	0.80	−0.65	2.49	1.33	0.249
Interaction of groups and time^†^						
Baseline	0	0				
2 weeks	−0.39	0.88	−2.12	1.33	0.20	0.656
4 weeks	−1.54	1.26	−4.01	0.92	1.50	0.220

*Note:* Covariates: Gender and *p* value: GEE model used with adjustment for covariates.

^†^Reference: Control group for the group effect, baseline values for the time effect, and baseline values of the control group for interactions.

^∗^ *p* < 0.05.

^∗∗^ *p* < 0.01.

**Table 6:** Effects of MBAP on participants' workplace bullying–victim and perpetrator aspects (*N* = 47).

	95% Wald CI					
	*B*	SE	Lower	Upper	Wald *χ*^2^	*p*
(i) Victim aspects						
Group^†^						
MBAP	2.62	4.38	−5.98	11.21	0.36	0.551
Time						
Baseline	0	0				
2 weeks	−1.42	2.63	−6.59	3.74	0.29	0.589
4 weeks	−2.39	2.70	−7.68	2.91	0.78	0.377
Interaction of groups and time^†^						
Baseline	0	0				
2 weeks	1.85	3.24	−4.50	8.20	0.33	0.568
4 weeks	−0.71	3.34	−7.26	5.84	0.05	0.832
(j) Perpetrator aspects						
Group^†^						
MBAP	5.56	2.32	1.01	10.11	5.73	0.017^∗^
Time						
Baseline	0	0				
2 weeks	0.15	0.75	−1.31	1.62	0.04	0.837
4 weeks	−0.42	0.72	−1.83	0.99	0.35	0.557
Interaction of groups and time^†^						
Baseline	0	0				
2 weeks	−0.12	1.56	−3.17	2.93	0.01	0.938
4 weeks	−1.96	1.95	−5.78	1.86	1.01	0.315

*Note:* Covariates: Gender and *p* value: GEE model used with adjustment for covariates.

^†^Reference: Control group for the group effect, baseline values for the time effect, and baseline values of the control group for interactions.

^∗^ *p* < 0.05.

## Data Availability

The data that support the findings of this study are available on request from the corresponding author. The data are not publicly available due to privacy or ethical restrictions.
